# Interpretable 2D Deep Learning for Alzheimer’s Detection from sMRI: A Lightweight Residual CNN Approach with Comprehensive Preprocessing and Stratified Data Partitioning

**DOI:** 10.3390/s26134100

**Published:** 2026-06-27

**Authors:** Vyshnavi Ramineni, Jun-Hyung Kim, Goo-Rak Kwon

**Affiliations:** Department of Information and Communication Engineering, Chosun University, Gwangju 61452, Republic of Korea; vyshnavi@chosun.ac.kr (V.R.); junkim@chosun.ac.kr (J.-H.K.)

**Keywords:** Alzheimer’s disease (AD), convolutional neural network (CNN), structural magnetic resonance imaging, Alzheimer’s Disease Neuroimaging Initiative (ADNI)

## Abstract

Neuroimaging is a promising modality for early AD detection, facilitating timely clinical intervention. This study proposes an enhanced deep learning framework that extracts critical AD biomarkers from structural MRI (sMRI) data acquired from the ADNI. Our novel CNN architecture integrates conventional convolutional layers with residual and skip connections for efficient feature extraction, achieving substantially lower computational cost than standard deep architectures such as VGG-16 (138 M), while remaining more parameter-intensive than highly compact architectures such as MobileNet and EfficientNet, which are designed explicitly for resource-constrained deployment. A comprehensive preprocessing pipeline converts 3D MRI scans into 2D slices through quality control (discarding slices with mean intensity < 5% of the maximum), bilinear resizing to 96 × 96 pixels, normalization using training-set statistics, and data augmentation. Stratified, subject-level data partitioning combined with robust statistical validation via bootstrapping demonstrates superior multiclass classification performance across AD, early and late MCI, and cognitively normal groups compared to state-of-the-art methods. Additionally, Grad-CAM-based interpretability maps were generated to highlight disease-relevant brain regions, confirming consistent activation around the hippocampus and temporal lobe.

## 1. Introduction

AD is the predominant causal factor contributing to the development of dementia. Alzheimer’s is defined as a degenerative pathology of the brain manifested by a range of debilitating symptoms, most notably memory loss and cognitive decline. These symptoms can reach a severity level that hinders daily functioning. AD is a prevalent condition, constituting 60–80% of all reported cases of [1] dementia. The high incidence of this condition underscores the extent of cognitive impairment. AD is widely recognized as a costly neurodegenerative disorder that imposes a substantial economic burden. In a study conducted in 2006 [2], approximately 26.6 million individuals worldwide were estimated to be afflicted by AD.

Given the increasing societal and economic ramifications of AD, its prognosis underscores the importance of preventive measures and interventions. Although certain indicators and manifestations of AD may bear resemblance to the cognitive decline associated with aging, it is essential to recognize that dementia, and in particular AD, are not indicative of a customary or inherent facet of the aging process. The clinical manifestations [3] of dementia exhibit a progressive pattern. Currently, definitive treatments for AD are lacking. The primary objectives include impeding the advancement of the disease, ameliorating behavioral complications, and enhancing overall life quality [4,5]. Existing pharmacological therapies have the potential to temporarily impede the relentless advancement of dementia by early detection of the characteristic indicators. Thus, the pursuit of enhanced therapeutic approaches, preventive strategies, and an ultimate remedy constitute a pivotal and enduring objective.

Recent studies have achieved substantial advancements in detecting and monitoring AD progression before the identification and utilization of biomarkers. Notably, brain imaging technologies are essential for detecting and visualizing the pathophysiological changes associated with AD throughout various periods, ranging from months to decades. In addition to amyloids, many other biomarkers that assess neurodegenerative processes have been utilized. The measures encompass the assessment of tau protein levels in the cerebrospinal fluid (CSF-tau), fluorodeoxyglucose positron emission tomography (FDG-PET) [6,7] and sMRI. Reliance on postmortem examinations underscores the need for continuous research to develop accurate, noninvasive diagnostic tools that facilitate early detection and intervention in living patients. Despite the extensive body of research dedicated to AD, there is an urgent need to develop reliable diagnostic tools because of the intricate and challenging nature of diagnosing and treating this condition.

The scale of the problem is significant. The World Alzheimer’s Report estimates that [8] diagnosed cases will rise from 55 million to 78 million by 2030. This condition is characterized by clinical symptoms, such as memory loss, confusion, and visuospatial abnormalities [9]. There are ongoing endeavors to enhance the early identification and diagnosis of this condition despite limited treatment options that rely on symptom monitoring alone. One such approach involves the discovery of specific CSF biomarkers [10]. However, this approach involves intrusive research that can potentially put patients at risk [11]. Advanced imaging modalities, such as positron emission tomography (PET) and MRI, play a crucial role in facilitating the identification of structural and molecular biomarkers associated with AD [12,13]. Due to its noninvasive nature, MRI has emerged as a crucial tool for comprehending the morphological and functional alterations in the brain that are associated with AD. Consequently, MRI is indispensable in clinical practice. There are challenges in integrating large-scale, multimodal, and high-dimensional data from new neuroimaging methods, which has led to a notable increase in the interest in integrative analysis using computational machine learning techniques.

Machine learning algorithms, although successful in illness classification, require labor-intensive and computationally intensive preprocessing techniques. A typical process involves four steps: feature extraction, feature selection, dimension reduction, and selection of a feature-based classification [14]. Thus, scientists are exploring deep learning (DL) algorithms as potential alternatives. DL algorithms are a specific type of representation learning technique that can generate optimal representations from unprocessed data without requiring prior feature selection [15,16,17]. Utilizing a complicated hierarchical structure with several tiers and sequential nonlinear transformations, DL [18,19] has demonstrated promise in various domains, such as medical imaging.

The CNN, with a widely used deep learning architecture, has garnered attention in the field of medical image analysis because of its notable performance in image categorization [20,21,22,23,24]. However, the CNN design can be further enhanced to attain a more realistic identification of AD. This study, motivated by the accomplishments of deep learning methods in the field of medical imaging, proposes an enhanced CNN to detect and classify AD by utilizing MRI images.

Despite notable advances in deep learning-based AD classification, several challenges remain. Existing high-performing architectures often involve substantial computational demands, which may limit practical deployment. Additionally, several studies have employed image-level rather than subject-level data partitioning, which can introduce data leakage and overestimate true generalization performance [18]. Interpretability also remains a concern, as the anatomical basis for model decisions is frequently not examined. The present study attempts to address these aspects by proposing a residual CNN that operates on 2D sMRI slices with subject-level stratified partitioning, 10-fold cross-validation, and Grad-CAM-based visualization. The key contributions include: (1) a relatively lightweight architecture; (2) subject-level data partitioning to mitigate leakage; (3) Grad-CAM analysis to examine anatomically relevant activations; and (4) multiclass classification across AD, EMCI, LMCI, and CN groups using the ADNI dataset.

The subsequent sections of this study are structured as follows. Section 2 provides an overview of the dataset and the proposed method, further presenting the evaluation criteria. The proposed architecture is presented in detail. The performance is evaluated and compared with other methods in Section 3 and further discussed. In Section 4, conclusions are drawn.

## 2. Materials and Methods

### 2.1. MRI Acquisition Protocol

The proposed model was tested using ADNI’s dataset. Of 600 subjects, 150 were diagnosed with AD, 150 with EMCI, 150 with LMCI, and 150 were cognitively normal (CN). The 3D MRI scans for each subject had dimensions of 256 × 256 × 170 pixels. Two-dimensional images were extracted from the axial, coronal, and sagittal planes, with blank or non-informative images automatically discarded. All remaining images were resized to 96 × 96 pixels using bilinear interpolation.

To ensure complete subject-level independence and avoid data leakage, the dataset was partitioned based on individual subjects rather than individual image slices. All slices derived from a single subject were kept within the same set. Specifically, a stratified split was performed in each diagnostic category (AD, EMCI, LMCI, CN) so that 70% of subjects were assigned to the training, 15% to the validation, and 15% to the testing. To robustly assess model performance, subject-level 10-fold cross-validation was performed exclusively within the 70% training partition. The validation set (15%) and test set (15%) were held out entirely prior to any fold iteration and were not used during cross-validation at any stage. Within the training partition, subjects were randomly divided into 10 equal folds at the subject level, ensuring that all slices from a single subject remained within the same fold. The model was trained on 9 folds and validated on the remaining fold, rotating until each fold had served as the validation fold once. Final model performance was reported on the held-out test set after the cross-validation procedure was completed.

In our preprocessing pipeline, raw 3D MRI scans (256 × 256 × 170 pixels) from the ADNI dataset are processed in several distinct steps. First, 2D slices are extracted from axial, coronal, and sagittal planes. To eliminate non-informative slices, we apply an intensity-based threshold: any slice with a mean pixel intensity below 5% of the maximum signal, or that fails a connectivity analysis for sufficient brain tissue, is automatically discarded. The remaining slices are then resized to 96 × 96 pixels using bilinear interpolation to support spatial consistency. Next, normalization is carried out by deducting the mean and dividing by the training set’s calculated standard deviation, ensuring that each image has zero mean and unit variance. In addition, to enhance the diversity of the training data and reduce overfitting, we employ data augmentation strategies including random rotations (±10°), horizontal flipping, and random intensity shifts. Standard skull stripping and MNI space registration were not applied, as the intensity-based quality filtering and slice-level normalization were deemed sufficient for the 2D classification approach. This choice reduces preprocessing complexity while maintaining classification performance, though it represents a limitation relative to volumetric methods. This detailed pipeline guarantees reproducibility and robust model training by thoroughly justifying all preprocessing decisions. This study adopts a 2D slice-based processing approach.

### 2.2. Data and Participants

The T1-weight MRI data, obtained from individuals enrolled in the ADNI over a span of 24 months, were examined. The study sample consisted of 150 individuals diagnosed with AD, 150 with EMCI, 150 with LMCI, and an additional 150 categorized as CN totaling 600 subjects. Demographic details for each group are provided in Table 1. Before performing quality assessment, the structural MRI images were subjected to preprocessing techniques. This study used data from ADNI, the main goal of which is to determine whether longitudinal MRI and PET imaging, along with other biological markers and comprehensive clinical and neuropsychological evaluations, can be integrated to track the progression of mild cognitive impairment and early-stage AD. The ADNI database “https://adni.loni.usc.edu (accessed on 20 January 2025)” was established in 2003 as a public–private partnership and can be accessed by approved researchers.

### 2.3. 2D Slice Extraction from 3D MRI Volumes

**Figure 1:**
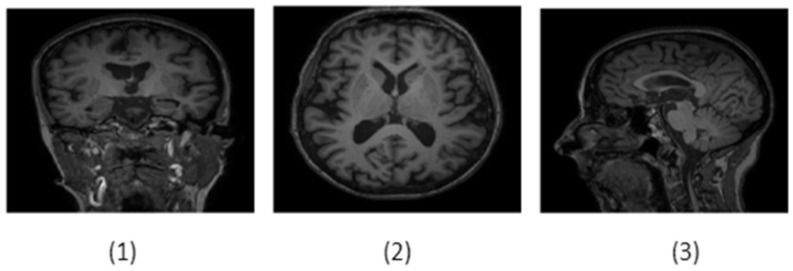
Three-plane brain MRI images from the ADNI database: (**1**) coronal; (**2**) axial; (**3**) sagittal.

The utilization of a 3D convolutional neural network is a rational decision for deep learning models because of the inherent volumetric characteristics of MRI data. The computational effort and time required to train 3D CNN models are considerably higher than those required to train 2D CNN models because of the high-dimensional input. A notable challenge arises from the limited scale of the prevailing medical datasets, hindering the effective training of a deep network from achieving generalization in intricate problem domains. In this study, the 3D MRI images used were unsuitable for direct application in 2D CNN models because of the number of dimensions. Two-dimensional slices were extracted directly from the raw 3D MRI volumes (256 × 256 × 170 voxels) along the sagittal, coronal, and axial planes without prior volumetric resampling, with the outermost sections containing no relevant brain information discarded. The initial and final slices lacking useful information were discarded. The slices were then normalized and resized, thereby obtaining images of mean zero and standard deviation one. The 2D convolutional neural network model was subsequently trained on randomly chosen axial, coronal, and sagittal patch slices. Figure 1 shows a collection of MRI slices from individuals with different cognitive states.

### 2.4. Network Architecture

The proposed CNN architecture integrates conventional convolutional layers with residual skip connections. Although 7 × 7 kernels are larger than the 3 × 3 kernels typical in lightweight models, they were selected in the initial convolutional layers to capture broader spatial context from MRI slices, where clinically relevant features such as hippocampal boundaries and ventricular enlargement span larger receptive fields. Despite the larger kernel size, the overall parameter count of 28.93 M remains substantially lower than VGG-16, justifying its relative computational efficiency. While more compact architectures such as MobileNet (~4 M parameters) and EfficientNet-B0 (~5 M parameters) exist, these models were originally designed for natural image classification and have not been optimized for neuroimaging tasks involving subtle morphological differences between diagnostic categories such as EMCI and LMCI. The proposed model with 28.93 M parameters achieves a balance between classification performance and computational efficiency that is appropriate for the complexity of 4-class AD classification from sMRI data. Furthermore, the relatively higher parameter count compared to the most lightweight models is justified by the need to capture fine-grained spatial features across multiple MRI planes that are critical for distinguishing transitional AD stages. Additionally, residual (skip) connections are incorporated to facilitate feature mixing, mitigate the vanishing-gradient problem, and capture both local and global spatial features, interleaved with max pooling, batch normalization, and dropout layers (dropout rate = 0.4). These residual blocks enable the network to learn complex representations by combining outputs from earlier layers with deeper layer activations, as detailed in our explicit architectural diagram, which includes the number of layers, filter dimensions, activation functions (ReLU), and dropout parameters. Furthermore, we compare the total trainable parameters, memory usage, and inference time of our proposed architecture with benchmark models such as VGG-16 and ResNet-50, substantiating our claims of lower computational costs.

Although 3D CNNs can directly analyze volumetric MRI data, our approach converts 3D scans into 2D slices to significantly reduce computational complexity and training time. This 2D processing allows us to leverage well-established image processing and data augmentation techniques—such as rotations, flips, and intensity shifts—while making efficient use of limited training data per class. While this conversion may lead to a partial loss of inter-slice spatial context, the substantial gains in computational efficiency and lower memory requirements justify this design choice. Each input underwent these procedures, thereby enabling the development of classifiers for both binary and multiclass categorization. Network performance was enhanced through the utilization of a collection of neurons that established connections between shift invariance, local connectivity, shared hyperparameters, and convolutional operation. The proposed comprehensive deep CNN framework aimed to detect numerous AD biomarkers. This framework utilizes the complete image volume as the input. In addition, in contrast to the suggested approach, we employed widely recognized CNN architectures, namely VGG-Net and ResNet, for the classification tasks. These models have demonstrated efficacy in a diverse array of applications including image classification, identification, labeling, and detection. In this study, we used cross-entropy as the dataset is balanced, enhancing model sensitivity. In this section, the proposed CNN architecture and associated methodologies employed in this study are described. Although the 2D-slice-based strategy significantly reduces computational burden, it inevitably removes inter-slice spatial dependencies present in full 3D volumes. This may lead to loss of contextual anatomical information; hybrid 2D architectures and slice-position encoding preserve volumetric context while maintaining efficiency.

#### 2.4.1. VGG_Net Model

**Figure 2:**
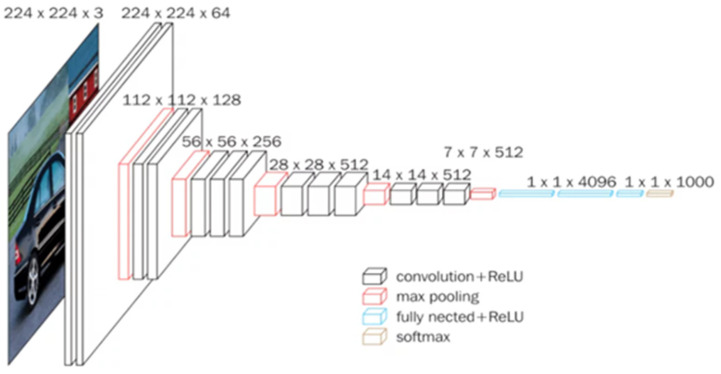
VGG-16 structural diagram.

The network, known as the proprietary CNN model, was first proposed by Simonyan and Zisserman [25]. The model was developed by the Visual Geometry Group (VGG) and trained on the ImageNet Large Scale Visual Recognition Challenge (ILSVRC) dataset. This dataset consists of 1.3 million photos, which were used for training, and an additional 50,000 images, which were used for validation. The dataset covers 1000 widely ranging classes. The VGG-13-19 model is a specific variety of VGG architecture distinguished by its 13–19 interconnected layers. The proposed model routinely demonstrated higher performance than other state-of-the-art models of the same period. The architectural design incorporates densely connected convolutional and fully connected layers, enabling sophisticated feature extraction. We employ average pooling to down-sample feature maps prior to applying the activation, Softmax, for classification. Using VGG-16 as our reference architecture, we then assess performance on the ADNI dataset by distinguishing AD. Figure 2 illustrates the VGG-16 architectural layout.

#### 2.4.2. ResNet

**Figure 3:**
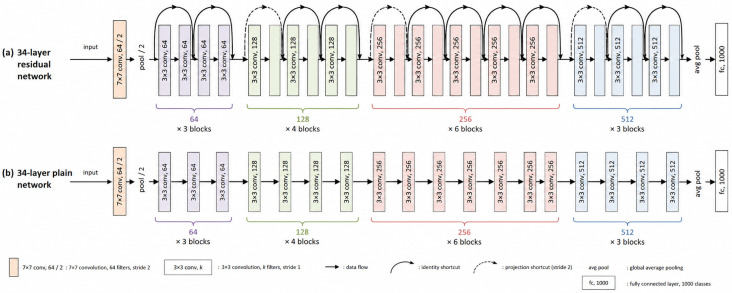
Architecture of ResNet pretrained on ImageNet: (**a**) plain network with 34 parameter layers; (**b**) residual network with 34 parameter layers.

ResNet quickly established itself as a leading architecture for categorization, localization, and recognition tasks during the ILSVRC competition [22]. To enhance cognitive ability, researchers investigated the necessity of incorporating additional layers into the neural network. During experimentation, researchers encountered a phenomenon known as degradation, wherein conventional models such as VGG exhibited a decline in performance instead of an improvement when the number of layers exceeded a certain threshold. Researchers have devised the concept of a residual function as a fundamental element within ResNet to address this challenge. In this study, the ResNet model employed the 50-layer non-bottleneck design shown in Figure 3. The present configuration comprises a series of connections that exhibit a progressive increase in size. These connections can be categorized into two types: identity links (A), which do not involve padding, and projection links (B), which employ convolutions with 1 × 1 filter (kernel) size. The categorization of AD was performed using the ResNet-50 model on the ADNI dataset. The diagram illustrates the fundamental structure of ResNet, with a simplified representation of 34 layers for clarity. Plain network with 34 parameter layers; Residual network with 34 parameter layers. Both networks were trained on the ImageNet dataset [26].

### 2.5. Proposed Methodology

**Figure 4:**
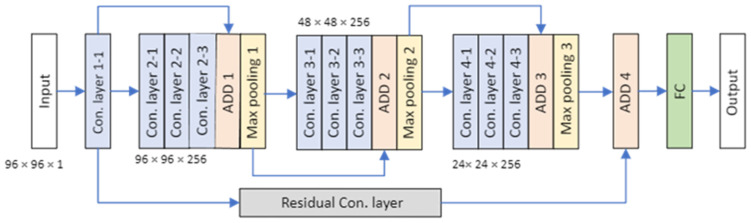
The layout of the proposed architecture. Con. Layer: Convolutional layer; Residual Con. Layer: residual convolutional layer; FC: fully connected layer.

Convolutional layers are fundamental to deep CNNs; by integrating activation functions, our deep CNN autonomously learns and extracts discriminative features from whole-brain MRI scans to enable accurate Alzheimer’s disease diagnosis. Figure 4 outlines the overall pipeline, which encompasses three main phases: CNN processing, slicing of 3D volumes, and scaling of brain volumes. Detailed layer configurations, including dimensions, are provided in Table 2 as the complete layer-by-layer architecture of the proposed model and Table S1 Layer-by-layer parameter count derivation for the proposed architecture. All max pooling layers use a 2 × 2 kernel with stride 2, halving the spatial dimensions at each stage. The three residual skip connections (ADD 1, ADD 2, ADD 3) are identity connections that add the input of each convolutional block directly to its output without spatial modification, as the spatial dimensions are preserved within each block. The global residual connection (ADD 4) spans the entire network from input to the final feature map. Since the input (96 × 96 × 1) and the final feature map (12 × 12 × 256) differ in both spatial dimensions and channel depth, a projection convolutional layer with a 1 × 1 kernel and stride 8 is used to match dimensions before the element-wise addition. A Global Average Pooling layer follows ADD 4 to reduce the spatial feature maps to a 1D vector before the fully connected output layer. Our method combines conventional convolutional layers with skip connections, allowing the model to learn and integrate features at multiple hierarchical levels from MRI images.

**Figure 5:**
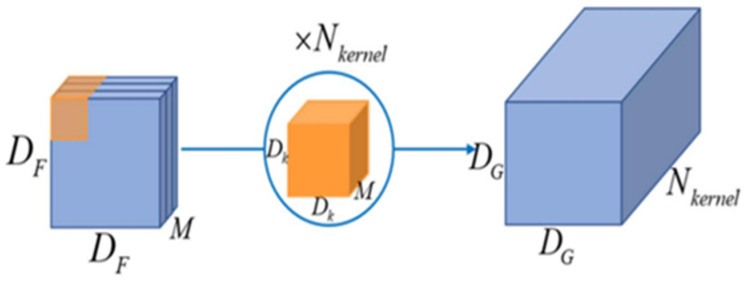
Illustration of the standard convolution architecture.

The CNN model is structured to refine feature extraction in a robust and efficient manner. Initially, convolution layers utilize 7 × 7 filters with 256 channels and employ ReLU activation to capture detailed spatial features from the input images. These convolution layers are followed by max pooling layers configured with specific strides and padding to down-sample the feature maps while preserving the most relevant information. To further stabilize training and reduce overfitting, batch normalization is applied together with dropout, set at a 0.4 rate. The architecture also incorporates residual blocks with skip connections, which enhance gradient flow and allow deeper feature integration by combining outputs from earlier layers with those from later layers. Finally, a fully connected layer serves as the output layer, mapping the extracted features to four distinct classes corresponding to the diagnostic categories in Alzheimer’s disease. The typical folding procedure is depicted in Figure 5, which also indicates the height and width of the square input feature map in spatial dimensions, and M and N denote the number of input and output feature map channels, respectively. In a convolutional layer, the input feature map I is convolved with the layer’s filters to produce the output G. This feature was removed from the conventional convolutional layer’s convolution kernel size. The height and width of the convolution kernel are indicated by $D_{K}$.

The basic convolution computation procedure is based on feature mapping. Map G can be highlighted using the following equation:

(1) $$G_{k,l,n}=\Sigma_{i,j,m}k_{i,j,m,n}\cdot I_{k+i-1.l+j-1,m,}$$

In Equation (1), k represents the convolution kernel, G represents the output feature map, and I denotes the input feature map. Here, *i* and *j* index spatial positions within the convolutional kernel, while *k* and *l* index spatial positions within the input and output feature maps, respectively. Additionally, M and N denote the channel indices for the input and output feature maps, respectively, allowing the convolution operation to consider multi-channel information.

The following formula calculates the number of trainable parameters F in a standard convolutional layer:

(2) $$F=M\times N\times D_{K}^{2}$$

where F is the total number of parameters, M is the number of input channels, N is the number of output channels, and $D_{K}$ is the spatial width and height of the convolutional kernel.

The following equation calculates the total computational cost G of a standard convolutional layer:

(3) $$G=M\times N\times D_{K}^{2}\times D_{F}^{2}$$

where G is the computing cost; here, $D_{F}$ is the spatial width and height of the input feature map, and M, N, and $D_{K}$ are as defined in Equation (2). The key distinction between Equations (2) and (3) is that the parameter count F depends only on the kernel and channel dimensions, whereas the computational cost G additionally scales with the spatial size of the input feature map $D_{F}^{2}$.

### 2.6. Implementation Details

**Figure 6:**
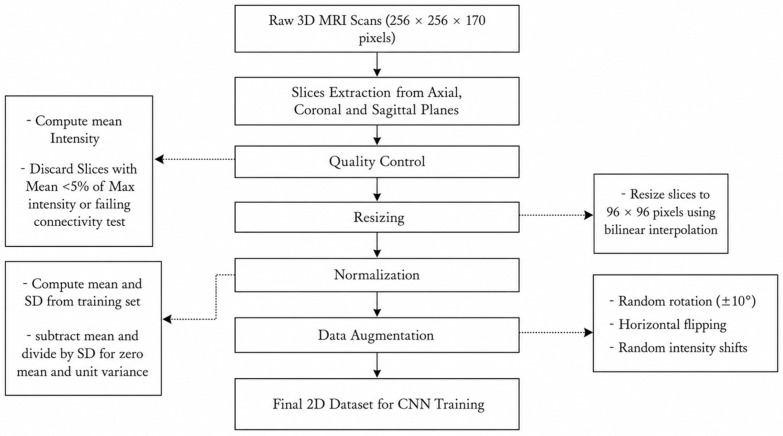
Preprocessing pipeline flowchart.

The experiments were executed on an NVIDIA RTX 3090 GPU running Ubuntu 20.04-x64 with Python 3.9.13, leveraging TensorFlow and Keras for model implementation. A two-dimensional CNN was developed using 2D slices extracted from 3D structural MRI scans Figure 6, with full reproducibility ensured through open-source preprocessing scripts and detailed data split configurations available from the corresponding author upon reasonable request. Categorical cross-entropy was used as the loss function, as it is appropriate for multiclass classification tasks involving more than two classes and is distinct from binary cross-entropy which applies only to binary classification scenarios. The use of categorical cross-entropy is further justified by the balanced class distribution across all four diagnostic categories shown in Table 3.

Weight initialization was performed using the Xavier (Glorot) method to maintain gradient stability. The preprocessing pipeline automatically extracted 2D slices from the axial, coronal, and sagittal views, discarded slices whose mean intensity was below 5% of the maximum and resized the remaining slices to 96 × 96 pixels via bilinear interpolation. Each image was normalized by subtracting the training-set mean and dividing by the training-set standard deviation, and data augmentation techniques including random rotations (±10°), horizontal flips, and random intensity shifts were used to enhance model generalization. For robust evaluation, performance metrics were computed with 95% confidence intervals derived from 1000 bootstrap resamples, and statistical significance was assessed via *p*-values when comparing against benchmark architectures. This integrated description and accompanying table consolidate our implementation and hyperparameter configurations in a concise format, ensuring that our methodology is both reproducible and statistically robust.

### 2.7. Performance Evalution

The terms true positive (TP), true negative (TN), false positive (FP), and false negative (FN) are used to describe the projected outcomes of the diagnostic tasks. A positive sample that is correctly predicted in advance is sometimes referred to as a “true-positive sample”; a negative sample that is correctly predicted or anticipated is sometimes referred to as a true negative. FP is used to denote the misclassification of a negative sample as a positive sample. In instances where symbol FN is present, there is a tendency for a positive sample to be erroneously classified as a negative sample. The diagnostic model was evaluated using a set of widely utilized measures, including the F1 score, accuracy, specificity, sensitivity, and precision. The accuracy (4) of a diagnostic test is determined by the correctly identified samples of all test samples. To compare our method with baselines more rigorously, we applied paired statistical tests including McNemar’s test for classification.

(4) $$Accuracy=\frac{TP+TN}{TP+TN+FP+FN}$$

As shown in Equation (5), specificity is calculated for the number of subjects that were correctly identified in Equation (6); sensitivity refers to the identification of the specified class in all positive samples. In the proposed method, in the context of AD patients, sensitivity is also called recall.

(5) $$Specificity=\frac{TN}{TN+FP}$$

As shown in Equation (7), precision is calculated as the ratio of true positive predictions to the total number of positive predictions.

(6) $$Sensitivity\;(Recall)=\frac{TP}{TP+FN}$$

(7) $$Precision=\frac{TP}{TP+FP}$$

As shown in Equation (8), the F1 score is the average value of precision and sensitivity.

(8) $$F1\text{-}Score=2\left(\frac{Precision\times Sensitivity}{Precision+Sensitivity}\right)$$

Multiclass classification performance was evaluated using a confusion matrix as shown in Table 4. This matrix displays the predicted versus the actual outputs for each class, allowing for a detailed assessment of the classifier’s performance. To support the interpretability claim, we generated Gradient-weighted Class Activation Mapping visualizations for correctly classified AD, LMCI, EMCI, and CN samples. The final convolutional layer’s feature maps were used to compute activation heatmaps, which were overlaid on their corresponding MRI slices. This analysis helps identify discriminative anatomical regions influencing model decisions, particularly the hippocampal formation, entorhinal cortex, and ventricular enlargement structures known to be associated with AD pathology.

## 3. Results and Discussion

### 3.1. Comparison with Previous Studies

Early CNN-based approaches for AD classification demonstrated the feasibility of using MRI data for automated diagnosis. Islam et al. [27] applied a patch-wise feature extraction technique on 416 MRI scans from the OASIS dataset, extracting 2D patches from axial, coronal, and sagittal planes and feeding them into a 2D DenseNet ensemble, achieving an accuracy of 93.18%. Jain et al. [28] selected the most informative 2D slices per subject using entropy-based comparison, which were subsequently fed into a VGG-16 transfer learning architecture, achieving a three-class accuracy of 95.73% on ADNI data. While VGG-based models offer a strong baseline, their large parameter count imposes significant memory and computational demands, limiting practical deployment in clinical settings.

To address the computational limitations of 2D approaches, several studies adopted 3D CNN architectures that process full MRI volumes. Hosseini-Asl et al. [9] applied a 3D convolutional autoencoder pretrained on the CAD dementia dataset and fine-tuned it on 210 ADNI subjects to extract AD biomarkers, achieving 89.1% accuracy for AD vs. NC classification. Liu et al. [29] proposed a multi-model framework combining a multi-task CNN and a 3D DenseNet operating on hippocampal regions, reporting 88.9% accuracy for AD vs. NC classification on 449 ADNI subjects.

Liu et al. [30] introduced a depthwise separable convolution-based CNN applied to OASIS MRI scans, reducing parameters to approximately 11K (three DSC layers) compared to the standard CNN baseline, while achieving 93.02% accuracy via GoogLeNet transfer learning. Xu et al. [31] proposed a modified TResNet model incorporating a Selective Kernel module operating on gray matter coronal MRI slices from 462 ADNI subjects, achieving 86.9% accuracy for binary AD vs. NC classification but only 63.2% for three-class classification, with 52 M parameters. These results suggest that parameter reduction does not always translate into improved multiclass performance, particularly when discriminating between closely related diagnostic categories such as EMCI and LMCI.

More recently, Vision Transformer (ViT)-based architectures have gained attention in medical image analysis due to their ability to model long-range spatial dependencies through self-attention mechanisms. However, transformers generally require large-scale training data to generalize effectively, and their application to small neuroimaging datasets remains challenging. Their computational demands also tend to exceed those of comparable CNN-based models, which further limits their adoption in resource-constrained settings.

Traditional machine learning methods have also been explored for AD classification. Lu et al. [32] applied a fuzzy k-nearest-neighbor classifier to 202 ADNI scans, achieving 88.90% accuracy. Zhang et al. [7] used a support vector machine with landmark-based features, achieving 85.71% accuracy for AD versus CN classification. Rani et al. [33] combined follow-up and cross-sectional data using a random forest classifier, achieving 91.2% accuracy for AD versus NC on 660 ADNI subjects. Nozadi et al. [34] classified AD and MCI patients using semantically parceled FDG-PET images from 660 ADNI subjects, applying a random forest classifier and reporting 72.5% accuracy for EMCI versus LMCI classification and 91.2% for AD versus CN classification. While this approach demonstrates the utility of PET-based biomarkers, it relies on an invasive and costly imaging modality compared to the sMRI-based approach adopted in the present study. Rukesh et al. [35] constructed a fully connected dense neural network for binary AD classification, outperforming six traditional machine learning methods. While these approaches are interpretable and computationally modest, they rely on handcrafted features and do not scale well to multiclass scenarios.

Across these approaches, three recurring limitations are evident. First, widely used architectures such as VGG-16 and 3D CNNs carry high parameter counts that limit deployment feasibility. Second, a number of studies do not apply subject-level data partitioning, which risks data leakage and inflated performance estimates [18]. Third, the anatomical basis for model decisions is rarely examined, limiting clinical trust in automated predictions. The proposed method attempts to address these aspects through a residual CNN with relatively fewer parameters, subject-level stratified partitioning with 10-fold cross-validation, and Grad-CAM-based interpretability analysis. Table 5 summarizes the comparison with published methods, and Table 6 compares performance against traditional machine learning approaches.

### 3.2. Discussion

For the prompt intervention and treatment of AD, a precise diagnosis is essential. Consequently, the focus of this study was to create computer-based tools, employing CNNs for medical image classification for the detection of Alzheimer’s disease in the initial stages. Despite the widespread use of CNNs, achieving efficient and optimal results is still challenging. This study explored an alternative approach for using CNN to improve MRI image categorization accuracy.

#### 3.2.1. Computational Complexity Analysis

Traditionally, efforts to improve classification performance have focused on increasing network depth and complexity. However, this study proposes a novel strategy to reduce the computational complexity and parameter count of the CNN, as shown in Table 7. The proposed model uses the same environment for these methods and is superior when considering parameters and time differences. Unlike earlier models which increase depth, the proposed approach addresses issues such as the vanishing-gradient problem. The network architecture includes the input, convolutional, and fully connected layers as three distinct layer types. The input layer processes N gray-level image patches, scaling them to 96 × 96 pixels and normalizing them before feeding them into the network. The convolutional layer incorporates skip connections, the Adam optimizer, dropout layers, batch normalization, and activation functions. Motivated by skip dropout layers, batch normalization, and activation functions, 33 filters were employed for the residual convolutional layer connections; the model consistently employs 256 filters at every stage, enabling weight sharing across different spatial regions of the images. The fully connected layer produces a 1D vector standing for the previously learned linear combination of neurons from layer one. Three CNN architectures, VGG, ResNet, and the proposed model, were used, each with its own characteristics. VGG networks, renowned for their consistent and regular architecture, are easy to customize but suffer from significant hardware requirements and many parameters. ResNet addresses these shortcomings by increasing layer depth to identify intricate patterns. The proposed model introduces the concept of feature mixing to mitigate the drawbacks associated with VGG and ResNet. First, although the proposed CNN is lightweight, computational trade-offs exist, as larger models may capture richer 3D context at the cost of higher memory usage.

#### 3.2.2. Subject-Level Data Partitioning

Another limitation is the reliance on a single dataset. Although ADNI is widely used, evaluation on independent cohorts such as OASIS or AIBL is necessary to establish generalizability across scanners and populations. Due to resource constraints, external validation could not be included in this study, but it will be prioritized in future work. Second, MRI scans in ADNI originate from multiple vendors and protocols, introducing unavoidable acquisition variability. Third, despite the balanced class distribution, subtle variance between EMCI and LMCI may still influence sensitivity in early-stage diagnostic groups. Finally, clinical deployment would require evaluation on multi-center and multi-scanner datasets.

#### 3.2.3. Model Interpretability

The heatmaps show the model consistently attends to regions corresponding to clinically relevant structures, including areas corresponding to the hippocampus and temporal lobe. However, precise anatomical localization would require registration to a standard brain template, which is a limitation of the current approach. AD samples exhibit high activation near medial temporal structures, whereas CN samples show diffuse low-intensity activations. Table 8 presents the numerical confusion matrix for the proposed model, and Table 9 summarizes the class-specific precision, recall, and F1-score for all four diagnostic categories.

**Figure 7:**
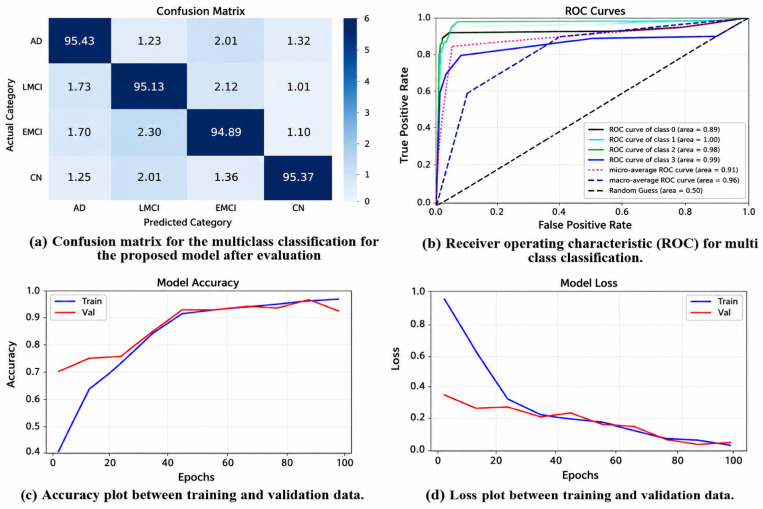
Visualization of model performance: (**a**) multi-class classification using confusion matrix, (**b**) ROC curve for AD, LMCI, EMCI, CN with classes, (**c**) accuracy graph on training and validation dataset, (**d**) loss plot for training/validation dataset.

**Figure 8:**
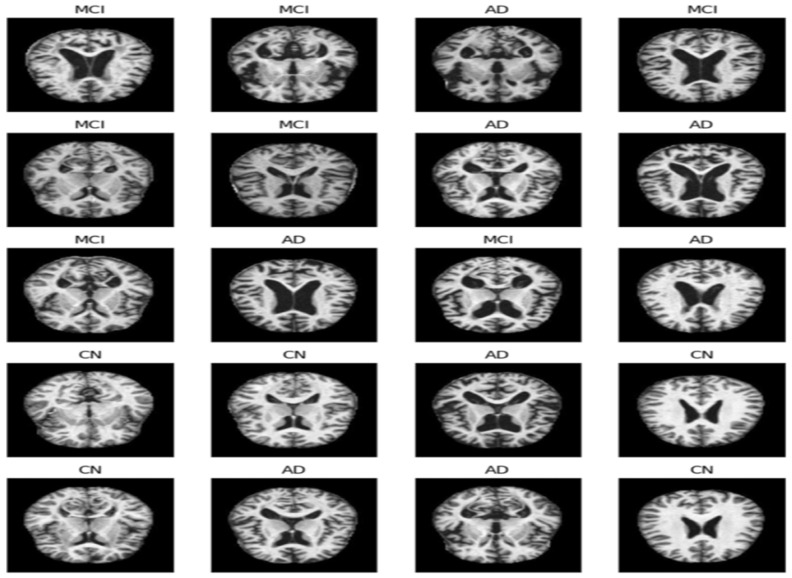
Representative MRI slices illustrating structural differences across Alzheimer’s disease stages.

**Figure 9:**
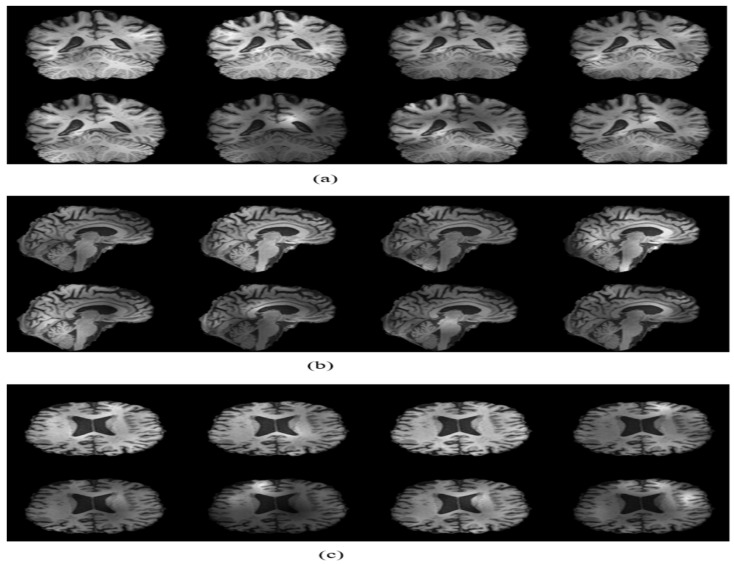
Grad-CAM visualizations overlaid on multi-view MRI slices. (**a**) Coronal, (**b**) sagittal, and (**c**) axial Grad-CAM maps generated by the proposed model.

The classification of EMCI and LMCI represents the most challenging aspect of multiclass AD diagnosis due to the subtle neuroanatomical differences between these two transitional stages. The proposed model achieved a recall of 94.89% for EMCI and 95.13% for LMCI, with F1-scores of 94.71% and 94.81%, respectively. The confusion matrix reveals that the primary source of misclassification occurs between EMCI and LMCI, where 2.30% of LMCI cases were misclassified as EMCI and 2.12% of EMCI cases were misclassified as LMCI. This pattern is consistent with findings in the literature, where discriminating between early and late MCI stages remains challenging even for experienced clinicians [18]. The confusion matrix displayed in Figure 7 was obtained using the proposed model for multiclass classification of four stages of Alzheimer’s; ROC for multi-class classification; and accuracy and loss plots of the training and validation dataset. The x and y axes of the confusion matrix represent the predicted and actual categories, respectively. The true-positive values are displayed on a scale ranging from 0 to 6. The percentage of truly predicted AD subjects was 95.43% for an AD diagnosis, and 95.13%, 94.89%, and 95.37% for LMCI, EMCI, and CN, respectively. All reported accuracy values are computed at the subject level, where predictions across all slices from a single subject are aggregated using majority voting before final classification. However, the values seen in the matrix are different from those falsely predicted in the actual class. Figure 8 presents axial brain MRI slices from subjects across four diagnostic groups: CN, MCI, and AD and an ROC curve plot for each class with it true positive rate (TPR) and false positive rate (FPR) to determine the model’s overall performance. CN subjects exhibit preserved cortical thickness and minimal ventricular enlargement. MCI subjects show early hippocampal atrophy, widening of temporal horns, and subtle cortical thinning, representing transitional neurodegeneration. AD subjects demonstrate pronounced medial temporal lobe atrophy, marked ventricular dilation, and diffuse cortical volume loss consistent with advanced Alzheimer’s pathology. These visual differences highlight the progressive anatomical changes that characterize the AD continuum. Figure 9 shows Grad-CAM maps [36] generated by the proposed model, highlighting the regions that most strongly contribute to Alzheimer’s disease classification. The heatmaps consistently focus on clinically relevant structures, including the hippocampus, medial temporal cortex, Para hippocampal gyrus, and enlarged ventricles. These spatial attention patterns demonstrate that the model prioritizes well-known Alzheimer’s biomarkers across different anatomical planes, supporting the interpretability and reliability of the framework. Comparative experiments revealed higher accuracy results for the proposed model, particularly in distinguishing groups at elevated risk of developing AD. Notably, the proposed model achieved an accuracy of 95.67%, indicating its potential for detecting patients with dementia and AD. Furthermore, interpretability analysis using Grad-CAM confirmed that the classifier relies on anatomically meaningful regions associated with AD progression, underscoring the clinical relevance of the learned features.

Future research will integrate multimodal biomarkers, explore transformer-based architectures for longitudinal AD progression, quantitatively assess Grad-CAM and anatomical region overlap using metrics such as the Dice coefficient or Intersection over Union to validate alignment with known AD biomarkers, and examine federated learning for cross-site privacy-preserving training.

#### 3.2.4. Ablation Study

To evaluate the contribution of each architectural component, an ablation study was performed by removing one element at a time while keeping all other settings fixed. As shown in Table 10, the full proposed model achieved the best overall performance (ACC = 95.67%, F1-score = 95.51%) with 28.93M parameters. Removing the residual/skip connections caused the largest drop (ACC = 91.63%), highlighting their role in gradient flow and feature integration. Replacing 7 × 7 kernels with 3 × 3 kernels nearly halved the parameter count (14.5 M) but reduced accuracy to 89.38%, indicating the importance of a larger receptive field. Excluding dropout and batch normalization led to further declines (ACC = 87.57% and 87.21%, respectively), confirming their role in regularization and stable training. These results show that each component contributes meaningfully to the model’s overall performance.

## 4. Conclusions

The results from this study demonstrate that the proposed CNN architecture, leveraging repeated convolutional blocks and residual skip connections, achieves high accuracy in classifying Alzheimer’s disease and its early cognitive impairment stages from structural MRI data. These findings significantly advance the field by providing an efficient, fully automated framework that not only reduces computational complexity but also enhances diagnostic precision in neuroimaging analysis. Despite these strengths, the study is limited by the reliance on 2D slice extraction from 3D scans, which may omit valuable inter-slice spatial information, and by the inherent variability in MRI acquisition protocols. Future research should focus on integrating hybrid 2D/3D approaches, improving preprocessing strategies such as cranial alignment, and incorporating additional clinical data to further validate and refine the model using independent datasets such as OASIS and AIBL, which would confirm the generalizability of the proposed approach across different scanner types and patient populations. Ultimately, our work paves the way for more robust and clinically applicable automated diagnostic tools in neurodegenerative disease detection.

## Figures and Tables

**Table 1:** Demographics distribution among subjects.

Class	No. of Participants	Age	Sex (M/F)
AD	150	76.6 ± 5.6	65/85
EMCI	150	78.09 ± 8.1	70/80
LMCI	150	76.34 ± 6.9	91/59
CN	150	79.56 ± 3.7	84/66

**Table 2:** Proposed architecture layers by size.

Layer Type	Layer Size	Filters	Stride	Source	Target
Con. Layer 1-1	7 × 7	256	1	96 × 96 × 1	96 × 96 × 256
Con. Layer 2-1	7 × 7	256	1	96 × 96 × 256	96 × 96 × 256
Con. Layer 2-2	7 × 7	256	1	96 × 96 × 256	96 × 96 × 256
Con. Layer 2-3	7 × 7	256	1	96 × 96 × 256	96 × 96 × 256
ADD 1 (Skip Connection)	-	-	-	96 × 96 × 256	96 × 96 × 256
Max pooling 1	2 × 2	-	2	96 × 96 × 256	48 × 48 × 256
Batch Normalization	-	-	-	48 × 48 × 256	48 × 48 × 256
Con. Layer 3-1	7 × 7	256	1	48 × 48 × 256	48 × 48 × 256
Con. Layer 3-2	7 × 7	256	1	48 × 48 × 256	48 × 48 × 256
Con. Layer 3-3	7 × 7	256	1	48 × 48 × 256	48 × 48 × 256
ADD 2 (Skip Connection)	-	-	-	48 × 48 × 256	48 × 48 × 256
Max pooling 2	2 × 2	-	2	48 × 48 × 256	24 × 24 × 256
Batch Normalization	-	-	-	24 × 24 × 256	24 × 24 × 256
Con. Layer 4-1	7 × 7	256	1	24 × 24 × 256	24 × 24 × 256
Con. Layer 4-2	7 × 7	256	1	24 × 24 × 256	24 × 24 × 256
Con. Layer 4-3	7 × 7	256	1	24 × 24 × 256	24 × 24 × 256
ADD 3 (Skip Connection)	-	-	-	24 × 24 × 256	24 × 24 × 256
Max pooling 3	2 × 2	-	2	24 × 24 × 256	12 × 12 × 256
Batch Normalization	-	-	-	12 × 12 × 256	12 × 12 × 256
Residual Con. Layer	1 × 1	256	8	96 × 96 × 1	12 × 12 × 256
ADD 4 (Global Skip Connection)	-	-	-	12 × 12 × 256	12 × 12 × 256
Global Average Pooling	-	-	-	12 × 12 × 256	256
FC	-	4	-	256	4

**Table 3:** Hyperparameter settings used in the experiment.

Hyperparameter	Value	Description
Learning Rate	0.0001	Step size for the Adam optimizer
Batch Size	32	Number of samples processed per gradient update
Number of Epochs	100 (with early stopping)	Maximum training iterations; early stopping based on validation loss
Dropout Rate	0.4	Dropout probability applied after pooling layers
Optimizer	Adam	Adaptive moment estimation optimizer
Weight Initialization	Xavier	Method to initialize weights for stable gradient flow
Loss function	Categorical cross-entropy	Appropriate for multiclass classification with more than two classes; distinct from binary cross-entropy

**Table 4:** True vs. predicted labels matrix (multiclass).

Prediction					
Actually	Classes	AD	LMCI	EMCI	HC
	AD	TP	$F_{AL}$	$F_{AE}$	$F_{AH}$
	LMCI	$F_{LA}$	TP	$F_{LE}$	$F_{LH}$
	EMCI	$F_{EA}$	$F_{EL}$	TP	$F_{EH}$
	CN	$F_{HA}$	$F_{HL}$	$F_{HE}$	TP

**Table 5:** Classification results using the proposed model and comparison with published methods.

Previous Studies	Dataset Collected (MRI)	No. of SUBJECTS	Task	Method	Parameters	ACC %	SEN %	SPEC %
Hosseini-Asl et al. [9]	ADNI	210	3-class	3D con autoencoder	76.22 M	89.10		
Islam et al. [27]	OASIS	416	4-class	2D DenseNet ensemble	65.32 M	93.18	93.00	
Jain et al. [28]	ADNI	150	3-class	VGG-16 + transfer learning	138 M	**95.73**		
Liu et al. [29]	ADNI	449	2-class	Multi-model CNN + 3D DenseNet	49.30 M	88.90	86.60	90.80
Liu et al. [30]	OASIS	490	3-class	Depthwise sep. CNN	29.80 M	93.02	83.21	75.32
Xu et al. [31]	ADNI	462	3-class	TResNet + Sk module	52 M	86.9	84.00	88.7
**This study**	ADNI	600	4-class	**Proposed method**	**28.93 M**	95.67	**94.16**	**97.34**

Note: Two-class (AD vs. CN); 3-class (AD vs. MCI vs. CN); 4-class (AD vs. EMCI vs. LMCI vs. CN).

**Table 6:** Classification results using the proposed model and comparison with traditional ML methods.

Previous Studies	Dataset	No. of Subjects	Task	Methodology	ACC %	SEN %	SPEC %
Lu et al. [32]	sMRI	AD-51, EMCI-43, LMCI-56, CN-52	4-class (AD/EMCI/LMCI/CN)	FKNN	88.90	86.60	90.80
Zhang et al. [7]	sMRI	AD-100, CN-115	Binary (AD/CN)	SVM	85.71	82.31	86.86
Rani et al. [33]	sMRI	660	Binary (AD/CN)	RF	91.20		
Nozadi et al. [34]	FDG-PET	AD-99, LMCI-189, EMCI-164, CN-208	4-class (AD/EMCI/LMCI/CN)	RF	72.5	79.2	69.9
Rukesh et al. [35]	sMRI	AD-58, MCI-60, CN-60	Binary (AD/MCI/CN)	FC neural network	87.50	83.33	91.70
**This study**	sMRI	600	**4-class (AD/EMCI/LMCI/CN)**	**Proposed method**	**95.67**	**94.16**	**97.34**

**Table 7:** Evaluating the performance of approaches in handling multiclass scenarios.

Methods	Parameters	Memory Usage (mb)	Time (ms)	ACC %	SEN %	SPE %	Precision %	F1-Score%
**ResNet**	25 million	100	40	95.17	**95.15**	96.49	94.77	94.75
**VGG 16**	138 million	250	60	91.27	92.31	95.63	91.39	91.32
**Proposed Model**	**28.9 million**	**50**	**10**	**95.67**	94.16	**97.34**	**95.43**	**95.51**

**Table 8:** Confusion matrix of the proposed model on the test set.

Class	Predicted AD	Predicted LMCI	Predicted EMCI	Predicted CN
**AD**	95.43	1.23	2.01	1.32
**LMCI**	1.73	95.13	2.12	1.01
**EMCI**	1.70	2.30	94.89	1.10
**CN**	1.25	2.01	1.36	95.37

**Table 9:** Class-specific performance metrics of the proposed model.

Class	Precision (%)	Recall %	F1-Score %
**AD**	95.33	95.43	95.38
**LMCI**	94.50	95.13	94.81
**EMCI**	94.53	94.89	94.71
**CN**	96.53	95.37	95.95
**Macro average**	**95.22**	**95.21**	**95.21**

**Table 10:** Ablation study of the model.

Model Variant	Parameters	ACC %	SEN %	SPE %	Precision	F1-Score
Full proposed model	28.93 M	95.67	94.16	97.34	95.43	95.51
Without residual/skip connections	26.1 M	91.63	86.35	91.85	90.60	89.20
3 × 3 kernels instead of 7 × 7	14.5 M	89.38	87.40	85.27	88.19	88.71
Without dropout (0.4)	28.93 M	87.57	86.72	85.94	85.74	86.31
Without batch normalization	28.93 M	87.21	86.59	88.25	84.45	86.93

## Data Availability

The data used in this study were obtained from the Alzheimer’s Disease Neuroimaging Initiative (ADNI) database “https://adni.loni.usc.edu (accessed on 20 January 2025)”. ADNI was launched in 2003 as a public–private partnership, led by Principal Investigator Michael W. Weiner, MD. The primary goal of ADNI has been to test whether serial MRI, PET, other biological markers, and clinical and neuropsychological assessment can be combined to measure the progression of MCI and early AD.

## Supplementary Materials

The following supporting information can be downloaded at https://www.mdpi.com/article/10.3390/s26134100/s1, Table S1. Layer-by-layer parameter count derivation for the proposed architecture.
