# Supplementary material for: Interpretable 2D Deep Learning for Alzheimer’s Detection from sMRI: A Lightweight Residual CNN Approach with Comprehensive Preprocessing and Stratified Data Partitioning
Source: Sensors (Basel). 2026 Jun 27;26(13):4100. doi: 10.3390/s26134100 (PMC13364039; doi:10.3390/s26134100)
Supplement: Supplementary file 1 [file sensors-26-04100-s001.zip › sensors-4365440-supplementary.pdf]

Supplementary Materials

Interpretable 2D Deep Learning for Alzheimer's Detection from sMRI: A Lightweight Residual CNN Approach with Comprehensive Preprocessing and Stratified Data Partitioning

Table S1. Layer-by-layer parameter count derivation for the proposed architecture.

Layer	Kernel	Input ch.	Output ch.	Formula	Parameters
Con. Layer 1-1	7×7	1	256	(7×7×1×256)+256	12,800
Con. Layer 2-1	7×7	256	256	(7×7×256×256)+256	3,211,520
Con. Layer 2-2	7×7	256	256	(7×7×256×256)+256	3,211,520
Con. Layer 2-3	7×7	256	256	(7×7×256×256)+256	3,211,520
Batch Normalization 1	—	256	256	2×256 (γ, β)	512
Con. Layer 3-1	7×7	256	256	(7×7×256×256)+256	3,211,520
Con. Layer 3-2	7×7	256	256	(7×7×256×256)+256	3,211,520
Con. Layer 3-3	7×7	256	256	(7×7×256×256)+256	3,211,520
Batch Normalization 2	—	256	256	2×256 (γ, β)	512
Con. Layer 4-1	7×7	256	256	(7×7×256×256)+256	3,211,520
Con. Layer 4-2	7×7	256	256	(7×7×256×256)+256	3,211,520
Con. Layer 4-3	7×7	256	256	(7×7×256×256)+256	3,211,520
Batch Normalization 3	—	256	256	2×256 (γ, β)	512
Residual Conv. Layer	1×1	1	256	(1×1×1×256)+256	512
Fully Connected Layer (FC)	—	256	4	(256×4)+4	1,028
Total					**28,919,556 (≈28.92M)**

Note: Convolutional layer parameters are computed as (kernel height × kernel width × input channels × output channels) + output channels (bias term). Batch normalization parameters correspond to the trainable scale (γ) and shift (β) terms, computed as 2 × the number of channels. The fully connected (FC) layer is computed as (input units × output units) + output units. Summing across all layers yields a total of 28,919,556 trainable parameters (≈28.92M), closely matching the reported value of 28.93M.
